# Supplementary material for: Unveiling the Bioactive Potential of the Invasive Jellyfish Phyllorhiza punctata Through Integrative Transcriptomic and Proteomic Analyses
Source: Biomolecules. 2025 Aug 4;15(8):1121. doi: 10.3390/biom15081121 (PMC12383608; doi:10.3390/biom15081121)
Supplement: Supplementary file 1 [file biomolecules-15-01121-s001.zip › Suplementary Material/FileS3/PEPFOLD4/XP_065069855_12790/clusters.html]

 PEP-FOLD clusters


Download archive of all models  here   

|  |  |  |
| --- | --- | --- |
| # | file | sOPEP |
| # cluster 1 - Size: 40 |  |  |
|  | XP\_065069855\_12790-model1 | -16.8021 |
|  | XP\_065069855\_12790-model1.1 | -16.7854 |
|  | XP\_065069855\_12790-model1.2 | -16.7403 |
|  | XP\_065069855\_12790-model1.3 | -16.7403 |
|  | XP\_065069855\_12790-model1.4 | -16.7164 |
|  | XP\_065069855\_12790-model1.5 | -16.7164 |
|  | XP\_065069855\_12790-model1.6 | -16.7032 |
|  | XP\_065069855\_12790-model1.7 | -16.7032 |
|  | XP\_065069855\_12790-model1.8 | -16.7032 |
|  | XP\_065069855\_12790-model1.9 | -16.7032 |
|  | XP\_065069855\_12790-model1.10 | -16.7032 |
|  | XP\_065069855\_12790-model1.11 | -16.7032 |
|  | XP\_065069855\_12790-model1.12 | -16.7032 |
|  | XP\_065069855\_12790-model1.13 | -16.7032 |
|  | XP\_065069855\_12790-model1.14 | -16.7032 |
|  | XP\_065069855\_12790-model1.15 | -16.7032 |
|  | XP\_065069855\_12790-model1.16 | -16.7032 |
|  | XP\_065069855\_12790-model1.17 | -16.7032 |
|  | XP\_065069855\_12790-model1.18 | -16.7032 |
|  | XP\_065069855\_12790-model1.19 | -16.7032 |
|  | XP\_065069855\_12790-model1.20 | -16.7032 |
|  | XP\_065069855\_12790-model1.21 | -16.7032 |
|  | XP\_065069855\_12790-model1.22 | -16.7032 |
|  | XP\_065069855\_12790-model1.23 | -16.7032 |
|  | XP\_065069855\_12790-model1.24 | -16.7032 |
|  | XP\_065069855\_12790-model1.25 | -16.7032 |
|  | XP\_065069855\_12790-model1.26 | -16.7032 |
|  | XP\_065069855\_12790-model1.27 | -16.7032 |
|  | XP\_065069855\_12790-model1.28 | -16.7032 |
|  | XP\_065069855\_12790-model1.29 | -16.7032 |
|  | XP\_065069855\_12790-model1.30 | -16.7032 |
|  | XP\_065069855\_12790-model1.31 | -16.7032 |
|  | XP\_065069855\_12790-model1.32 | -16.7032 |
|  | XP\_065069855\_12790-model1.33 | -16.7032 |
|  | XP\_065069855\_12790-model1.34 | -16.7032 |
|  | XP\_065069855\_12790-model1.35 | -16.7032 |
|  | XP\_065069855\_12790-model1.36 | -16.7032 |
|  | XP\_065069855\_12790-model1.37 | -16.7032 |
|  | XP\_065069855\_12790-model1.38 | -16.6612 |
|  | XP\_065069855\_12790-model1.39 | -16.5945 |
| # cluster 2 - Size: 29 |  |  |
|  | XP\_065069855\_12790-model2 | -16.7797 |
|  | XP\_065069855\_12790-model2.1 | -16.7797 |
|  | XP\_065069855\_12790-model2.2 | -16.7797 |
|  | XP\_065069855\_12790-model2.3 | -16.7797 |
|  | XP\_065069855\_12790-model2.4 | -16.7797 |
|  | XP\_065069855\_12790-model2.5 | -16.7797 |
|  | XP\_065069855\_12790-model2.6 | -16.6752 |
|  | XP\_065069855\_12790-model2.7 | -16.6752 |
|  | XP\_065069855\_12790-model2.8 | -16.6752 |
|  | XP\_065069855\_12790-model2.9 | -16.6752 |
|  | XP\_065069855\_12790-model2.10 | -16.6752 |
|  | XP\_065069855\_12790-model2.11 | -16.6752 |
|  | XP\_065069855\_12790-model2.12 | -16.6752 |
|  | XP\_065069855\_12790-model2.13 | -16.6752 |
|  | XP\_065069855\_12790-model2.14 | -16.6752 |
|  | XP\_065069855\_12790-model2.15 | -16.6752 |
|  | XP\_065069855\_12790-model2.16 | -16.6752 |
|  | XP\_065069855\_12790-model2.17 | -16.6752 |
|  | XP\_065069855\_12790-model2.18 | -16.6752 |
|  | XP\_065069855\_12790-model2.19 | -16.6752 |
|  | XP\_065069855\_12790-model2.20 | -16.6752 |
|  | XP\_065069855\_12790-model2.21 | -16.6752 |
|  | XP\_065069855\_12790-model2.22 | -16.6752 |
|  | XP\_065069855\_12790-model2.23 | -16.6752 |
|  | XP\_065069855\_12790-model2.24 | -16.6508 |
|  | XP\_065069855\_12790-model2.25 | -16.6508 |
|  | XP\_065069855\_12790-model2.26 | -16.6508 |
|  | XP\_065069855\_12790-model2.27 | -16.6334 |
|  | XP\_065069855\_12790-model2.28 | -16.5289 |
| # cluster 3 - Size: 11 |  |  |
|  | XP\_065069855\_12790-model3 | -16.7201 |
|  | XP\_065069855\_12790-model3.1 | -16.7201 |
|  | XP\_065069855\_12790-model3.2 | -16.7201 |
|  | XP\_065069855\_12790-model3.3 | -16.7201 |
|  | XP\_065069855\_12790-model3.4 | -16.6693 |
|  | XP\_065069855\_12790-model3.5 | -16.6693 |
|  | XP\_065069855\_12790-model3.6 | -16.6693 |
|  | XP\_065069855\_12790-model3.7 | -16.6518 |
|  | XP\_065069855\_12790-model3.8 | -16.6518 |
|  | XP\_065069855\_12790-model3.9 | -16.6518 |
|  | XP\_065069855\_12790-model3.10 | -16.5881 |
| # cluster 4 - Size: 8 |  |  |
|  | XP\_065069855\_12790-model4 | -16.6919 |
|  | XP\_065069855\_12790-model4.1 | -16.6919 |
|  | XP\_065069855\_12790-model4.2 | -16.6919 |
|  | XP\_065069855\_12790-model4.3 | -16.6919 |
|  | XP\_065069855\_12790-model4.4 | -16.6471 |
|  | XP\_065069855\_12790-model4.5 | -16.6471 |
|  | XP\_065069855\_12790-model4.6 | -16.5986 |
|  | XP\_065069855\_12790-model4.7 | -16.5972 |
| # cluster 5 - Size: 7 |  |  |
|  | XP\_065069855\_12790-model5 | -16.6739 |
|  | XP\_065069855\_12790-model5.1 | -16.6739 |
|  | XP\_065069855\_12790-model5.2 | -16.6739 |
|  | XP\_065069855\_12790-model5.3 | -16.6739 |
|  | XP\_065069855\_12790-model5.4 | -16.6739 |
|  | XP\_065069855\_12790-model5.5 | -16.5695 |
|  | XP\_065069855\_12790-model5.6 | -16.5695 |
| # cluster 6 - Size: 5 |  |  |
|  | XP\_065069855\_12790-model6 | -16.6074 |
|  | XP\_065069855\_12790-model6.1 | -16.6074 |
|  | XP\_065069855\_12790-model6.2 | -16.6074 |
|  | XP\_065069855\_12790-model6.3 | -16.6074 |
|  | XP\_065069855\_12790-model6.4 | -16.5985 |
|  |  |  |
